# HRD Testing of Ovarian Cancer in Routine Practice: What Are We Dealing With?

**DOI:** 10.3390/ijms241310497

**Published:** 2023-06-22

**Authors:** Tatiana Kekeeva, Yulia Andreeva, Alexander Tanas, Alexey Kalinkin, Svetlana Khokhlova, Tatiana Tikhomirova, Alexandra Tyulyandina, Anatoly Popov, Maria Kuzmenko, Mikhail Volkonsky, Natalia Chernorubashkina, Valeria Saevets, Vadim Dmitriev, Valentina Nechushkina, Olga Vedrova, Sergei Andreev, Sergey Kutsev, Vladimir Strelnikov

**Affiliations:** 1 Laboratory of Epigenetics, Research Centre for Medical Genetics, Moskvorechie St., 1, 115522 Moscow, Russia; 2 Department of Pathology, Russian Medical Academy of Continuing Professional Education, 125993 Moscow, Russia; 3 Oncological Department of Medical Treatment, National Medical Research Center for Obstetrics, Gynecology and Perinatology Named after V. I. Kulakov, 117997 Moscow, Russia; 4 N.N. Blokhin National Medical Research Center of Oncology, Ministry of Health of Russia, 115478 Moscow, Russia; 5 National Medical Research Center of Surgery Named after A. Vishnevsky, Department of Antitumor Drug Therapy, 117997 Moscow, Russia; 6 Department of Oncology, Radiology and Radiotherapy, Tyumen State Medical University, 625023 Tyumen, Russia; 7 Day Hospital No. 1, Moscow Municipal Oncological Hospital No. 62, 143423 Moscow, Russia; 8 Department of Surgical Methods of Treatment No. 9, State Budgetary Healthcare Institution Regional Oncological Dispensary, 664035 Irkutsk, Russia; 9 Gynecological Oncology Department, Chelyabinsk Regional Clinical Centre for Oncology and Nuclear Medicine, 454087 Chelyabinsk, Russia; 10 Department of Theoretical Surgery, Belgorod National Research University, 308015 Belgorod, Russia; 11 Department of Oncology, Diagnostic Radiology and Radiotherapy, Privolzhsky Research Medical University, 603005 Nizhniy Novgorod, Russia; 12 Astrazeneca Pharmaceuticals LLC, 123112 Moscow, Russia

**Keywords:** homologous recombination deficiency, *BRCA* mutation, genomic instability score, tumor purity, pathological response

## Abstract

Assessment of homologous recombination deficiency (HRD) status is now essential for ovarian cancer patient management. The aim of our study was to analyze the influence of ethnic variations, tumor purity, and neoadjuvant chemotherapy (CT) on the determination of HRD scores as well as to evaluate feasibility of HRD testing with the Amoy HRD Focus Assay in routine clinical practice. The HRD status, including the *BRCA* status and genomic scar score (GSS), was analyzed in 452 ovarian cancer specimens. The successful rate of HRD testing was 86% (388/452). The *BRCA* mutational rate was 29% (114/388); 252 samples (65%) were classified as HRD-positive. Our data demonstrate the feasibility of internal HRD testing by the AmoyDx HRD Focus Panel for high-grade serous ovarian cancer (HGSOC), showing results similar to other methods. The HRD rate in the Russian population is very similar to those of other European populations, as is the *BRCA* mutation frequency. The most substantial contribution to HRD level diversity is testing criteria depending on intrahospital arrangements. The analysis shows that biallelic *BRCA* alterations had higher GSS compared with those with monoallelic inactivation, consistent with positive HRD status. The study indicates that grades 1–2 of the pathological response caused by chemotherapy affect HRD scores and suggests controlling for tumor purity of 40% or more as a critical factor for GSS measurement.

## 1. Introduction

Ovarian cancer (OC) is the seventh most commonly diagnosed cancer among women in the world [1]. The standard treatment for newly diagnosed advanced epithelial ovarian cancer is surgical cytoreduction and systemic platinum–taxane combination chemotherapy. Unfortunately, most patients with advanced ovarian cancer do not have an effective treatment option to reduce the risk of progressive disease after first-line chemotherapy due to many factors, including performance status, frailty index, duration of initial treatment response, adverse events, histology, location and burden of disease, and genetics [2,3]. Currently, there are many new drugs and molecular mechanisms under development and being tested in ongoing clinical trials aimed at evaluating their efficacy in the treatment of ovarian cancer [4,5].

Poly ADP–ribose polymerase (PARP) inhibitors have efficacy as single agents in the treatment of recurrent ovarian cancer and as a maintenance therapy after patients have had a response to platinum-based therapy [2,6,7,8]. Homologous recombination deficiency (HRD) is a common feature of high-grade serous ovarian cancer (HGSOC) [9,10,11]. Recent clinical trials demonstrated its predictive potential by evaluating patient responses to platinum-based therapies and PARP inhibitors [6,7,8]. According to The Cancer Genome Atlas data, HRD-positive status is the most prevalent alteration in ovarian cancer (69%) [9].

Developing reliable methods to determine the HRD status of tumors is important for optimizing the clinical benefits of PARP inhibitors. HRD detection methods can be divided into three categories based on their features. One is based on detecting mutational profiles, such as HRDetect [12]. These methods require whole-exome sequencing or whole-genome sequencing data to achieve accurate mutation profiles. Another category analyzes the gene expression profile [13]. The third category identifies CNV features, including the genomic scar score (GSS), Amoy, Myriad HRD, and foundation HRD methods [14]. HRD is remarkable for the frequent copy number alterations occurring at the regional or whole-chromosome level. Quantification of large-scale structural variants is used as an indicator of the HRD phenotype, including telomeric allelic imbalance (large allelic imbalances extending to the telomere), large-scale transition (number of transitions between large regions of different allelic states or chromosomal breaks between adjacent regions of >10 MB), and loss of heterozygosity (large regions displaying somatic loss of one haplotype). The Amoy model displayed more than 97.0% sensitivity in detecting BRCA-deficient events, and the GSS model identified patients that could benefit from PARP inhibitors, as the GSS-positive group had a longer progression-free survival (PFS) (9.4 versus 4.4 months) than the GSS-negative group [14].

Accurate detection of HRD may also be influenced by some analytical and preanalytical factors such as tumor purity and neoadjuvant chemotherapy (CT) effect. Approximately 20% of patients with ovarian cancer are treated with neoadjuvant platinum-based chemotherapy followed by interval debulking surgery [15]. Chemo-sensitive clones are thought to disappear, and chemo-resistant clones are thought to remain after chemotherapy. Hence, the effects of CT on the HRD rate of pretreated tumors must be evaluated. Although each of these main characteristics of HGSOC has been investigated in several studies, no reports have examined the association between HRD and grades of pathological response caused by chemotherapy and how these factors changed before and after chemotherapy. Furthermore, the distribution and frequency of *BRCA* and other HRR mutations demonstrate significant geographical and ethnic variations, so the mutational rate contributes to the ethnic HRD rate. The aim of our study was to analyze the influence of these factors on the determination of HRD scores as well as to evaluate the feasibility of HRD testing with the Amoy HRD Focus Assay in routine clinical practice.

## 2. Results

**Figure 1:**
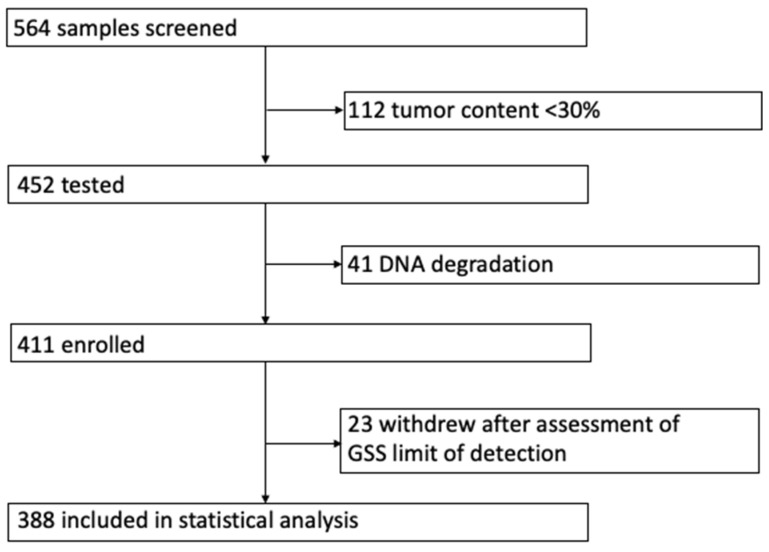
Study profile.

Simultaneous analysis of the whole coding regions and exon–intron boundaries of *BRCA*1/2 and analysis of 24,000 SNPs followed by GSS calculation was performed. Among the 564 ovarian cancer specimens referred for HRD analysis, 112 were excluded from the genetic testing, as they did not reach the minimum tumor content required by the manufacturer instructions (above 30%). The remaining 452 cases were included in the testing. However, 41 samples did not pass the bioinformatic filter due to DNA degradation (mean depth after UMI filtration for SNPs region ≥ 200). We performed the analysis of HRD patterns in a total of 411 tumors (Figure 1).

### 2.1. Impact of Tumor Purity

**Figure 2:**
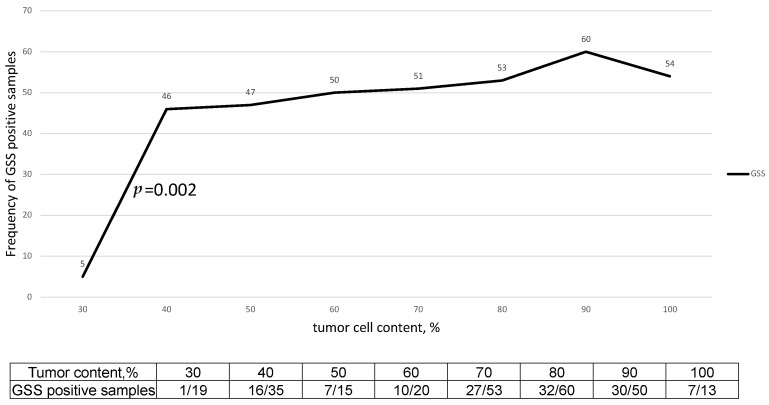
Impact of tumor purity on the frequency of GSS-positive samples. The significant difference between 30% tumor and 40% tumor was detected by a chi-square test (*p* = 0.002).

We analyzed the influence of tumor purity on the determination of the GSS score. To systematically analyze the effect of tumor purity, we excluded *BRCA* mutant cases (BRCAmut) and divided *BRCA* wild-type (BRCAwt) samples into 8 groups of 30%, 40%, 50%, …, up to 100% of tumor cell content (Figure 2). In a correlation analysis of the positive GSS score frequency and tumor purity, we detected a significant 9-fold decrease of GSS positive samples for the group of 30% tumor content and other groups (*p* = 0.002). 

We thus withdrew 23 samples (19 wtBRCA and 4 mutBRCA) with 30% tumor content from the following statistical analysis. The clinical and pathological characteristics of the 388 tumors are summarized in Table 1.

### 2.2. HRD and BRCA Rates

The successful rate of HRD testing was 86% (388/452). The HRD-positive rate was 56% (252/452) among all tested samples and 65% (252/388) excluding undetermined cases.

The results of *BRCA1/2* and GSS analysis are reported in Table 2. In all, 252 samples (65%) were classified as HRD-positive, including 114 BRCAmut and 138 BRCAwt cases with GSS ≥ 50, whereas 136 samples were identified as HRD-negative (no *BRCA* mutations and no GSS positive status).

Furthermore, 114 tumors had pathogenic or likely pathogenic *BRCA*1/2 variants, of which 72 were located at *BRCA1* and 40 at *BRCA2*; 2 tumors had mutations in both genes. The occurrences of the eight recurrent (≥2 samples) variants are presented in Table 3. The other PVs were detected once. The full list of PVs/LPVs identified is shown in Supplementary Table S1.

### 2.3. Analysis of Pre- and Post-CT Tumor Samples

**Figure 3:**
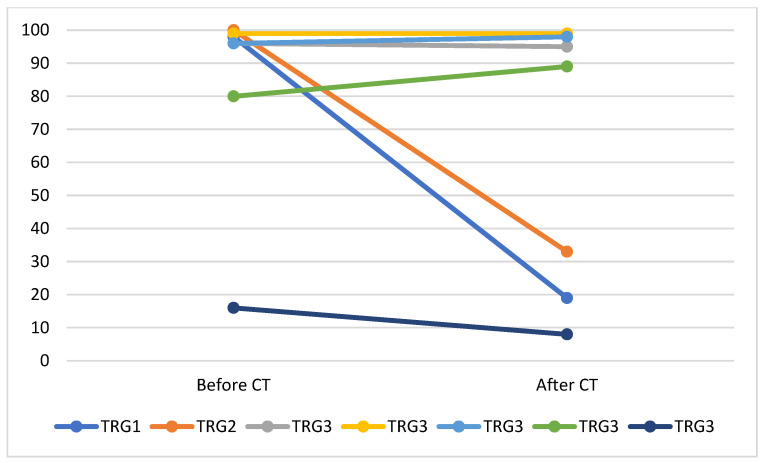
GSS scores changes before and after CT.

HRD positivity rates were 55% (29/53) for treated tumors and 65% (213/327) for nontreated tumors. Additionally, we measured GSS status in the BRCAwt paired tumors from seven patients obtained before and after chemotherapy (CT). Tumor regression grading (TRG0–TRG3) was used to measure the response to therapy in post-treatment tissue [16]. One case had a grade 1 response (no or minimal tumor) post-CT; the GSS score descended from 98 to 19 after CT. A second case had a grade 2 response (partial tumor response); the GSS score descended from 100 to 33 after CT. Additionally, 5 cases had no response (grade 3), and the GSS scores were unchanged: 96 to 95, 99 to 99, 96 to 98, 80 to 89, and 16 to 8 before and after treatment, respectively (Figure 3).

### 2.4. Biallelic versus Monoallelic BRCA1/2 Mutations

The criteria for BA alteration classification were fulfilled for 52 *BRCA*-positive tumors. We detected BA status in 36/37 (97%) of BRCAmut tumors with high GSS scores and in 1/37 (3%) of tumors with low GSS scores. BRCAmut MAs were identified in 8/15 (53%) of high-GSS specimens and 7/15 (47%) of low-GSS samples (*p* < 0.00001).

In addition, 101/114 *BRCA* mutant samples were GSS-positive, demonstrating high correlation; 13 samples had *BRCA* PVs but no genomic instability (Table 4).

## 3. Discussion

We analyzed the influence of tumor purity on the determination of GSS scores. A significant decrease in high GSS scores was detected for the group of 30% tumor content compared to other groups of 40%, 50%, 60%, and up to 100% tumor content. These data as well as TCGA data support the idea that unbiased determination of the GSS score is feasible for samples with a tumor purity of 40% or more, while lower purity relates to a systematic downshift of the HRD rate [9]. The percentage of tumor cells is generally estimated by a pathologist, but data concerning the reproducibility of these estimates are not encouraging, and they depend on subjective opinions [17]. We chose a cutoff point of 40% tumor cells, which is close to the manufacturer’s recommended diagnostic threshold of 30% but reduces the risk of overestimation of tumor cell content that may lead to an unreliable result.

The HRD status, including tBRCA and the GSS score, was analyzed. The *BRCA* mutational rate was 29% (113/388), including 10% of Russian founder mutations (39/388). East Slavonic populations (the Russians, the Ukrainians, and the Belarusians) comprise 90% of the tested patients. *BRCA1* c.5266dup is the most frequent germline PV in Russian ovarian cancer patients. Other recurrent *BRCA1* PVs (c.4035del, c.181T>G, c.1961del, c.68_69del, c.3756_3759del, and c.3700_3704del) also have wide distributions in Russia, accounting together for up to 50% of all *BRCA*1/2 PV cases [18]. We detected c.5266dup in 34/388 cases, c.1961del in 3/388, c.4035del in 1/388, and c.68_69del in 1/388, which is 35% of all *BRCA* PVs and lower than we anticipated. We suppose that the reduced frequency of well-known PVs is the result of routine genetic screening performed before HRD testing for the early detection of targetable genetic alterations. However, PVs not included in the routine screening were discovered with expected frequencies, in particular *BRCA2* c.5286T>G, recently identified in Russian ovarian cancer patients as a new founder mutation [19]. The *BRCA* testing in our study has some limitations. First, we could not collect blood samples, so the germline or somatic mutation status was not determined. However a previous investigation has demonstrated that 80% of *BRCA*-mutated Russian HGSOC patients have germline mutations [18]. A more important limitation of the blood samples’ absence is the failure to test for *BRCA* large rearrangements (LRs). In Russia, *BRCA* LRs are known to be rare (1.8%) [18].

We compared HRD rates from different hospitals and identified three trends impacting intrahospital HRD rates. First, clinicians recommend testing for all primary HGSOC cases. Second, doctors consider HRD testing after *BRCA* founder mutation screening, which resulted in an artificially low HRD rates of 35% (11/31). Third, doctors consider testing patients with young age at diagnosis, multiple primary cancers, or other cancers associated with hereditary syndromes. In this instance we observed higher HRD rates up to 71% (17/24) for some hospitals. We can conclude that the HRD rate in the Russian population is very similar to those of other European populations, with the same being the case for the BRCA frequency. The most substantial contribution to HRD level diversity is testing criteria depending on intrahospital arrangements.

Neoadjuvant chemotherapy, followed by interval tumor reductive surgery, is increasingly used for patients with advanced extrauterine HGSOC because upfront complete gross resection is not feasible for many patients. However, reliable predictive indicators of the response to CT are still lacking. Changes evident in tumor samples from interval surgery are not well understood, but histologic grading could be more reliable than clinical evaluations for assessing the response to treatment and even for identifying predictive features for the risk of progression and death [20].

Histologically, features of chemotherapy-related changes are regarded as indicators of regression in tumors. Chemotherapy induces changes in gene expression and alters the mutational profile [21]. To evaluate the notion that patients with HGSOC could be better selected for HRD testing based on chemotherapy-related changes, we compared the GSS statuses of paired tumors from seven patients obtained before and after chemotherapy. Tumors with a complete or partial pathological response demonstrated decreased GSS scores after CT. Tumors not responding to treatment had unaltered GSS scores. It has been established that tumor cells deficient in homologous recombination repair and having genomic instability are eliminated by CT because they are sensitive to platinum agents. The residual cell clones do not harbor genomic instability. The effects of CT on the downshift of GSS scores of pretreated tumors are underreported. However, most of the TRG 1–2 samples do not reach the minimum tumor content required for HRD testing, and rare cases do not affect markedly the statistical frequency. In the entire treated cohort, minimal pathological response was observed in 40% of cases; partial response 50% of cases; and complete pathological response in 12% cases [20]. At the same time, according to our records the most frequent post-CT specimens are TRG3 tumors because of FFPE selection with more than 30% tumor cells. Our data show HRD positivity rates of 55% (29/53) and 65% (213/327) for treated and nontreated tumors, respectively. Still, in routine HRD testing we do not recognize the CT effect on the HRD rate, and hence the testing of separate TRG2 tumors could results in falsely low GSS scores.

Here, we analyzed the association between *BRCA* mutation status and GSS score. The percentage of tumors with high GSS scores was significantly higher in tumors harboring *BRCA1/2* PVs compared to tumors with BRCAwt (139/274 vs. 101/113, *p* < 0.00001).

Separation BA and MA hits represent an important feature of mutations in tumor suppressor genes, as the former hit type is associated with loss of function, while the latter one is associated with retention of function. Applying the approach to separate BA and MA alterations described in the Methods section, we found that the percentage of BA alterations of all *BRCA1/2* alterations was 37/51 (73%), including 36 specimens with high GSS scores and 1 sample with a low GSS score. MAs were observed in 8/15 and 7/15 *BRCA* tumors with high GSS scores and with low GSS scores, respectively. Notably, biallelic inactivation of *BRCA1/2* was associated with higher HRD scores, while MA had no impact on it. In line with Knudson’s two-hit hypothesis and the strong dependence of GSS scores on the allelic hit type observed in this study, biallelic but not monoallelic alterations should be considered primarily for the therapeutic profit of HRD. Indeed, clinical benefit was almost exclusively observed in patients who had biallelic *BRCA* and a high HRD score, confirming the absence of a functional homologous repair system [22].

The detailed description of 13 *BRCA* mutants with the absence of genomic instability is shown in Table 4. The low GSS scores may be related to previous chemotherapy (6 cases), near-cut-off values of GSS between 40 and 50 (5 samples), levels of tumor purity close to the limit of detection of 40% (2 samples), and all these factors together. These factors can be classified as preanalytical and analytical factors. Still, we observed five tumors without previous treatment, with sufficient tumor content, and with unequivocally low GSS scores. It is important that the loss of the alternative allele was not detected for all six cases, suggesting monoallelic *BRCA* inactivation. In line with genetic considerations, our data also show that absence of the second *BRCA* allele hit also influences the probability of association with low GSS scores. Further understanding of the molecular basis of true monoallelic *BRCA* alterations not related to methodology is required; however we can conclude they are rare events in HGSOC.

The most reputed HRD test is the Myriad MyChoice CDx. Recently, three investigations demonstrated the feasibility of the AmoyDx HRD Focus Panel and showed high concordances between Amoy and Myriad MyChoice [23,24,25]. Weichert and colleagues demonstrated the following agreements between the AmoyDx HRD Focus Panel and myChoice CDx for 98 tumors: positive percent agreement (PPA) 88.0%, negative percent agreement (NPA) 75.0%, and overall percent agreement (OPA) 81.6% [23]. According to Fumagalli et al., the OPA was 87.8%, the PPA was 83.3%, and the NPA was 100% for 74 tested samples [25]. In our study the Myriad MyChoice CDx report was available for three patients. Our data show complete concordance of the two tests for these specimens, in accordance with the abovementioned studies.

Applying the HRD Focus Panel, we identified 65% of tumors as HRD-positive. Recently Rempell et al. published TCGA data demonstrating HRD-positive status in 69% of ovarian cancer samples measured by whole-exome sequencing [9]. These data are in line with the incidences reported in the PAOLA1 (255/447, 57%), PRIMA (247/416, 59%), VELIA (214/339, 63%), and ARIEL3 (236/343, 69%) trials, as detected by the Myriad MyChoice and FoundationOne CDx systems [2,6,7,8]. Here it is worth mentioning that we did not take into account undetectable specimens in the clinical trial data. For comparison we show prevailing HRD rates with account taken of undetermined HRD cases: our study—240/452 (53%), PAOLA1—255/537 (48%), PRIMA—247/487 (51%), VELIA—214/382 (56%), and ARIEL3—236/375 (63%). Accounting for all enrolled cases is common in clinical trials but results in a decreased rate compared to a cohort of evaluated cases only. Moreover, this approach depends on a successful testing rate and could distort the actual value due to differing quality of FFPE.

Our data demonstrate the feasibility of internal HRD testing with the AmoyDx HRD Focus Panel for HGSOC, showing results similar to those of previous studies. The HRD rate in the Russian population is very similar to those of other European populations, and the same is true for *BRCA* mutation frequency. The most substantial contribution to HRD level diversity is testing criteria depending on intrahospital arrangements. The analysis shows that biallelic *BRCA* alterations had higher GSS compared with those with monoallelic inactivation, consistent with positive HRD status. The study indicates that grades 1–2 of pathological response caused by chemotherapy affect HRD scores and suggests controlling for tumor purity of 40% or more as a critical factor for GSS measurement.

## 4. Material and Methods

### 4.1. Study Cohort

A total of 564 patients with a histological diagnosis of HGSOC were recruited from Russian hospitals between April 2021 and December 2022. Eligible patients were women aged 18 years or older.

### 4.2. Histopathological Specimens

The histological subtype and tumor, node, and metastases staging were reviewed by a pathologist. For each selected sample, manual macrodissection was performed, and sections with more than 30% content of tumor cells were obtained for molecular analyses.

### 4.3. Evaluation of Neoadjuvant Chemotherapy

The effect of neoadjuvant chemotherapy was assessed based on the system for tumor regression grading (TRG). Scoring was carried out on a single H&E-stained section. A single block that showed the least response to chemotherapy was selected. The amount of viable tumor tissue was assessed. A complete regression was defined as mainly regression, with few irregularly scattered individual tumor cells or cell groups (all measuring less than 2 mm) (TRG1), or no residual tumor being identified (TRG0). A partial response was defined as multifocal or diffuse regression-associated fibro-inflammatory changes, with viable tumor tissue ranging from diffuse sheets, streaks, or nodules to extensive regression with multifocal but easily identifiable residual tumor tissue (TRG2). Minimal tumor response was defined as mainly viable tumor tissue with minimal regression-associated fibro-inflammatory changes limited to a few foci (TRG3). As a guide, >95% of tumor tissue should be viable for a score of 3, and <5% for a score of 1.

### 4.4. Sample Preparation and NGS Analysis

DNA was isolated using the GeneRead DNA FFPE Treatment Kit (Qiagen, Hilden, Germany). HRD evaluation was performed with an HRD Focus Assay (CE-IVD) provided by AmoyDx (AmoyDx, Xiamen, China), following the manufacturer’s instructions. The test kit is based on the Halo-shape Annealing and Defer-Ligation Enrichment (HANDLE) system technology, which is an improved molecular inversion probe (MIP), to capture the target gene region. A unique molecular identifier (UID) is introduced to both ends of each DNA fragment to trace back to the original template for error correction. The probe contains an extension arm and a ligation arm which are complementary to the target gene region. First, the extension arm and ligation arm are anchored to the target gene region, and the DNA is extended from the extension arm to the ligation arm using DNA polymerase. Next, the nicks are connected with the ligase to generate the circular products. The remaining linear probes and single-strand and double-strand nucleic acid are digested with the exonuclease. Finally, universal PCR amplification is performed to enrich the target libraries. Briefly, 100 ng of DNA extracted from representative slides of tumor tissue blocks was used for library preparation and then sequenced on an Illumina NextSeq platform. This assay allowed the simultaneous analysis of SNVs and indels in the whole coding regions and exon–intron boundaries of *BRCA1*/*BRCA2* and estimated a genomic scar score (GSS) based on the analysis of 24,000 SNPs. A GSS equal or higher than 50 was indicative of HRD positivity. A positive HRD status result is due to either the presence of a pathogenic (PV)/likely pathogenic (LPV) variant in the *BRCA1/2* genes or a positive GSS status. The bioinformatic algorithm applied for the NGS data analysis was Andas AmoyDx (version 1.1.1). Recently, the authors of the software described the learning-based GSS algorithm in detail [14]. Tissue sample NGS data were analyzed blindly with regard to the germline/somatic status.

### 4.5. Biallelic versus Monoallelic Alterations for BRCA Mutant

According to Knudson’s hypothesis, tumor suppressor genes require the inactivation of both alleles to drive cancer [26]. Thus, dependent on whether a single allele or both alleles were affected, we classified the hit type of a mutation as either biallelic (BA) or monoallelic (MA). Assuming that at least half of the reads of the corresponding alleles included mutations, we used the threshold of 70% for the variant allele frequency (VAF). Specimens were corrected for tumor purity, and only tumors with 70% or more of tumor cell content were analyzed. Alterations were classified as BA if a mutation with VAF ≥ 70% was detected in a specimen with 70% or more tumor cells. Alterations were classified as MA if a mutation with VAF < 60% was detected in a specimen with 70% or more tumor cells. Other *BRCA* mutant specimens with 60% ≥ VAF ≥ 70% were classified as unavailable.

### 4.6. Variant Classification

Variants were annotated according to Human Genome Variation Society nomenclature. Variants were classified as PV or LPV (collectively termed pathogenic) according to the American College of Medical Genetics and Genomics recommendations and the CanVIG-UK Consensus Specification for Cancer Susceptibility Genes guidelines.

### 4.7. Statistical Analysis

Statistical analysis was carried out using the SPSS Statistics 25 software. A chi-square test was used for data comparison of categorical variables; *p*-values < 0.01 were considered statistically significant.

## Figures and Tables

**Table 1:** Patient clinical characteristics. CT—chemotherapy.

**Age, years, median**	57 (31–85)
**Diagnosis**	388
**Epithelial ovarian cancer**	365 (94%)
**Fallopian tube cancer**	13 (3%)
**Primary peritoneal cancer**	10 (3%)
**Tumor obtained before CT treatment**	327 (84%)
**Tumor obtained after CT treatment**	53 (14%)
**No information about CT**	8 (2%)

**Table 2:** Summary of genetic alterations identified in HGSOC (n = 388).

**Samples tested**	452
**Undetectable HRD status**	64
**tBRCA mutant**	114/388 (29%)
**tBRCA1 mutant**	72 (18.6%)
**tBRCA2 mutant**	40 (10.3%)
**Both genes**	2 (0.5%)
**tBRCA mutant GSS high**	101/114
**tBRCA mutant GSS low**	13/114
**tBRCA wild type**	
**GSS high**	138 (36%)
**GSS low**	136 (35%)
**HRD high**	252/388 (65%)

**Table 3:** Recurrent pathogenic or likely pathogenic variants in *BRCA1* and *BRCA2* genes identified.

Gene	Variant Name	Occurrence
*BRCA1*	NM_007294.4:exon20:c.5266dup:p.(Q1756Pfs*74)	34 (8.8%)
*BRCA2*	NM_000059.3:exon11:c.5286T>G:p.(Y1762*)	7 (1.8%)
*BRCA1*	NM_007294.4:exon11:c.1961del:p.(K654Sfs*47)	3 (0.8%)
*BRCA2*	NM_000059.3:exon11:c.3847_3848del:p.(V1283Kfs*2)	3 (0.8%)
*BRCA1*	NM_007294.4:intron18:c.5152+1G>T	3 (0.8%)
*BRCA1*	NM_007294.4:exon11:c.1687C>T:p.(Q563*)	2 (0.5%)
*BRCA2*	NM_000059.3:exon11:c.2808_2811del:p.(A938Pfs*21)	2 (0.5%)
*BRCA1*	NM_007294.4:exon20:c.5251C>T:p.(R1751*)	2 (0.5%)

**Table 4:** Characteristic of BRCAmut samples with low GSS. VAF—variant allele frequency, LOH—loss of heterozygosity, NA—not applicable.

Case Number	Pretreatment	Tumor Cell Content, %	GSS Score	BRCA Variant	VAF, %	Biallelic Inactivation
1314	yes	60	11	NM_007294.4:exon20:c.5266dup:p.(Q1756Pfs*74)	44	no
423	yes	90	45	NM_007294.4:exon11:c.1961del:p.(K654Sfs*47)	49	no
1322	no	70	46	NM_007294.4:exon20:c.5266dup:p.(Q1756Pfs*74)	46	no
266	no	80	23	NM_007294.4:exon11:c.2157dup:p.(E720Rfs*6)	18	no
1518	no	45	9	NM_007294.4:exon20:c.5266dup:p.(Q1756Pfs*74)	51	NA
335	no	40	43	NM_007294.4:intron18:c.5152+1G>T	55	no
1621	no	85	29	NM_000059.3:exon11:c.3637G>T:p.(E1213*)	56	no
3319	no	80	7	NM_000059.3:exon11:c.6082_6086del:p.(E2028Kfs*19)	44	no
135	yes	40	41	NM_007294.3:exon2:c.53T>C:p.(M18T)	62	NA
292	yes	50	23	NM_007294.4:exon20:c.5266dup:p.(Q1756Pfs*74)	50	NA
332	yes	60	46	NM_000059.3:exon11:c.6591_6592del:p.(E2198Nfs*4)	64	NA
478	yes	50	36	NM_007294.4:exon20:c.5266dup:p.(Q1756Pfs*74)	48	NA
425	no	50	1	NM_007294.4:exon2:c.66dup:p.(E23Rfs*18)	52	NA

## Data Availability

Research data may be provided upon reasonable request.

## Supplementary Materials

The supporting information can be downloaded at: https://www.mdpi.com/article/10.3390/ijms241310497/s1.
